# 小细胞肺癌组织中LncRNA AK09398的表达及与预后的关系研究

**DOI:** 10.3779/j.issn.1009-3419.2016.11.06

**Published:** 2016-11-20

**Authors:** 斌 柳, 晓琴 刘, 业 杨, 俊 葛, 静 李

**Affiliations:** 1 610041 成都，四川省肿瘤医院肺部肿瘤病区 Department of Pulmonary Tumor Ward, Sichuan Cancer Hospital, Chengdu 610041, China; 2 610041 成都，四川省肿瘤医院第二门诊部 the Second Outpatient Department, Sichuan Cancer Hospital, Chengdu 610041, China

**Keywords:** 肺肿瘤, LncRNA AK09398, 预后, Lung neoplasms, LncRNA AK09398, Prognosis

## Abstract

**背景与目的:**

小细胞肺癌（small cell lung cancer, SCLC）约占肺癌的15%，由于较早发生血行及淋巴转移，SCLC患者的预后差，其具体机制尚不明确。研究发现LncRNA（noncoding RNA, ncRNA）在肿瘤的发生、发展、转移、凋亡中起着重要的作用。本研究旨在探讨LncRNA AK09398在SCLC组织标本中的表达及临床意义。

**方法:**

通过qRT-PCR法检测LncRNA AK09398在118例临床资料完整的SCLC肺癌组织及正常肺组织标本中的表达，分析其表达与患者临床病理特征及预后的关系。

**结果:**

LncRNA AK09398在肺癌癌组织中的平均表达水平为（7.813±0.373），与癌旁组织（1.782±0.116）及正常肺组织（1.209±0.200）比较，差异有统计学意义（*F*=58.41, *P* < 0.001）。LncRNA AK09398在SCLC组织标本中的表达较癌旁组织明显增高。LncRNA AK09398的表达与患者的年龄、性别无关，差异无统计学意义（*P*均 > 0.05）；与疾病分期、淋巴结转移及远处转移、化疗敏感性及生存状态明显相关，差异有统计学意义（*P*均 < 0.05）。高表达LncRNA AK09398患者的总生存时间及无进展生存时间均较低表达者明显缩短；*Cox*多因素回归模型分析提示，LncRNA AK09398的表达、远处转移及临床分期是SCLC独立的预后因素（*P* < 0.05）。

**结论:**

LncRNA AK09398参与调节SCLC的发生发展，可能作为潜在的SCLC预后评估的分子标志物。

## Abstract (en)

**Background and objective:**

Small cell lung cancer (SCLC) accounts for approximately 15% of lung cancer. The poor prognosis of SCLC patients is attributed to the early blood and lymph node metastasis. However, the mechanism of SCLC is still unclear. Recent studies show that LncRNA plays an important role in the development, metastasis, and apoptosis of tumor. Thus, the present study aimed to investigate the expression and clinical significance of LncRNA AK09398 in SCLC.

**Methods:**

Quantitative reverse transcription polymerase chain reaction (qRT-PCR) was used to detect the expression of LncRNA AK09398 in 118 cases of SCLC and non-cancerous tissues. qRT-PCR was also utilized to analyze the relationship between the expression and clinical pathological characteristics and prognosis of SCLC.

**Results:**

The expression of LncRNA AK09398 in lung cancer tissues was (7.813±0.373). This value was significantly increased in paracarcinoma tissues (1.782±0.116) and normal lung tissues (1.209±0.200). The difference was statistically significant (*F*=58.41, *P* < 0.001). AK09398 expression was positively correlated with disease stage, lymph node and distant metastasis, chemosensitivity, and survival status (*P* < 0.05). However, AK09398 expression was not associated with gender and age (*P* > 0.05). *Kaplan-Meier* analysis demonstrated that high expression level of AK09398 contributed to poor overall survival (*P* < 0.001) and progression-free survival (*P* < 0.001). The multivariate survival analysis also indicated that AK09398 expression, disease stage, and distant metastasis could be independent prognostic markers.

**Conclusion:**

LncRNA AK09398 is involved in the development of SCLC and can be used as a molecular marker to evaluate the prognosis of SCLC.

肺癌为一种恶性程度较高的肿瘤性疾病，其病死率居各种恶性肿瘤之首，发病率逐年上升^[1]^。小细胞肺癌（small cell lung cancer, SCLC）是支气管肺癌中未分化癌分型，约占全部肺癌的15%。由于较早发生较强的侵袭性和早期发生血行及淋巴转移，大多数患者就诊时已发生转移，中位生存期不足1年，5年生存率不足5%^[2]^。SCLC的治疗主要以化疗为主。虽然SCLC患者早期对一线化疗方案（足量EP方案：依托泊式或伊立替康+顺铂/卡铂）敏感，但由于极易出现多药耐药（multidrug resistance, MDR）现象而导致治疗失败。因此，寻找有效的早期诊断及疗效预测标志物是临床亟待解决的难题。

随着非编码RNA（noncoding RNA, ncRNA）研究的不断深入，发现ncRNA（主要包括microRNA、长链非编码RNA等）在肿瘤的的发生、发展、转移、凋亡中起着重要的作用，虽然基因组分析发现了大量的LncRNAs，但目前只有少部分LncRNAs被广泛用于研究，这些LncRNAs具有作为肿瘤诊断、预后分子标志物的潜力，其表达水平与多种肿瘤的临床病理特征以及预后相关^[3-5]^。研究其在SCLC发生发展中的作用及相关性将为SCLC的早期诊断及治疗提供新突破。课题组前期通过LncRNA芯片发现LncRNA AK09398在SCLC耐药细胞株中的表达较敏感细胞株明显增高，提示LncRNA AK09398可能与SCLC的发生发展相关。本研究通过qRT-PCR方法检测LncRNA AK09398在SCLC患者肺癌组织及其对应的正常肺组织标本中的表达情况，并对患者的临床病理特征进行分析，揭示LncRNA AK09398在SCLC中的可能作用，并探讨LncRNA AK09398在SCLC早期诊断及预后评估中的潜在作用。

## 1 材料与方法

### 1.1 标本来源

118例SCLC患者化疗前组织标本均来源于2011年1月-2015年12月本院肿瘤科、胸外科及呼吸内科行手术及穿刺活检或支气管镜活检的组织标本，其中53例SCLC原发病灶及癌旁组织（癌旁组织距离癌组织达5 cm以上），65例患者癌组织取自支气管镜或计算机断层扫描（computed tomopraphy, CT）引导下穿刺活检标本，65例非肺癌患者的正常肺组织取自各种原因导致的肺外伤的患者，所有患者病例资料完整，术前均未行放化疗。男性55例，女性63例，年龄30岁-83岁，平均年龄为（56±7.7）岁，体能状态（performance status, PS）评分根据病程记录和入院记录进行评价，统一采用美国东部肿瘤协作组（Eastern Cooperative Oncology Group, ECOG）评分标准，PS 0分-1分患者70例，PS≥2分48例，吸烟患者79例，有恶性肿瘤家族史24例，局限期50例，广泛期68例。所有病例均经过病理学检查确诊为SCLC，均接受EP（依托泊苷+顺铂/卡铂）方案化疗及全脑预防性放疗，本研究经本院伦理委员会批准，所有患者均签署知情同意书。随访：全部118例患者出院后均随访，随访方式为电话随访和门诊随访，随访内容包括一般情况、临床症状及影像学检查。随访起点为手术或病理活检日期，末次随访时间为2016年6月30日，至随访截止日，存活病例49例，死亡病例69例，无失访病例。

### 1.2 方法

#### 1.2.1 Real-time PCR分析LncRNA AK09398的表达水平

采用Trizol（Invitrogen, Grand Island, NY）方法提取组织标本中总RNA。将提取的总RNA，逆转录反应参照AMV逆转录试剂盒说明，在20 μL体系中加2 μg总RNA进行cDNA的合成。Real-time PCR采用2×SYBR Green PCR Master Mix，取适量cDNA作为摸板，引物浓度0.4 mol/L，15 μL体系进行扩增，每个待测样本设置3个平行样，根据目标基因设计合成相应上下游引物进行PCR扩增，以GAPDH作为内参照。PCR反应在定量PCR反应仪上进行。三次独立实验后得到的数据运用公式RQ=2^-ΔΔCt^的方法进行分析。

### 1.3 统计学方法

采用SPSS 13.0统计软件进行数据分析。LncRNA AK09398在癌组织、癌旁正常组织中的表达差异采用方差分析。LncRNA AK09398在癌组织和癌旁组织中的表达及其与各临床病理参数之间的关系分析使用*Chi-Square*检验；采用*Kaplan-Meier*法分析LncRNA AK09398的表达与生存时间及预后的关系，应用*Cox*比例风险模型分析影响非小细胞肺癌（non-small cell lung cancer, NSCLC）预后的因素，以*P* < 0.05为差异具有统计学意义。

## 2 结果

### 2.1 LncRNA AK09398在SCLC组织标本中的表达

LncRNA AK09398在肺癌癌组织中的平均表达水平为（7.813±0.373），与癌旁组织（1.782±0.116）及正常肺组织（1.209±0.200）比较，差异有统计学意义（*F*=58.41, *P* < 0.001）（图 1）。

**1:**
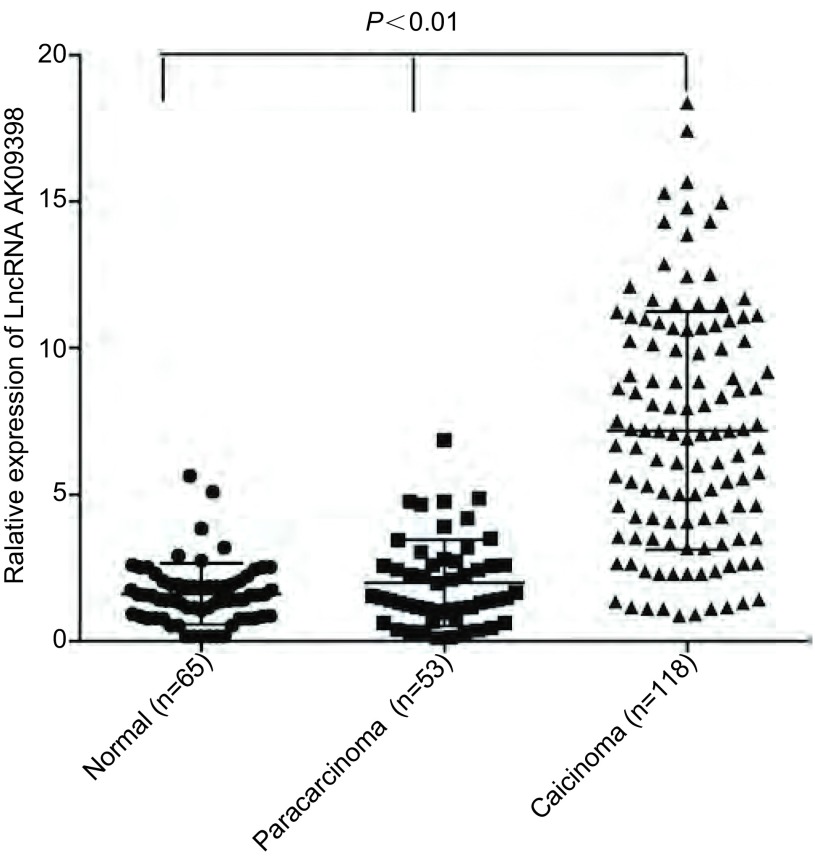
qRT-PCR法检测LncRNA AK09398在非肿瘤组织及SCLC组织中的表达 The expression of LncRNA AK09398 were measured in nontumor tissues and SCLC tissues by qRT-PCR

### 2.2 SCLC组织标本中LncRNA AK09398的表达及其与临床病理特征的关系

118例SCLC患者中男性55例，女性63例；年龄30岁-83岁，中位年龄56岁， < 56岁52例，≥56岁66例。广泛期68例，局限期50例；淋巴结转移者70例，无淋巴结转移者48例；远处转移68例，无远处转移50例。截止研究工作完成，存活病例49例，死亡病例69例。根据LncRNA AK09398平均表达水平（7.183），将SCLC患者分为LncRNA AK09398高表达组66例及低表达组52例，分析LncRNA AK09398的表达与患者年龄、性别、疾病分期、化疗敏感性、转移及患者的生存期的关系，结果发现LncRNA AK09398的表达与患者的年龄、性别无关，差异无统计学意义（*P*均 > 0.05）；与疾病分期、淋巴结及远处转移、化疗敏感性及生存状态明显相关，差异有统计学意义（*P*均 < 0.05）（表 1）。

**1:** SCLC患者组织标本中LncRNA AK09398的表达与病理特征的关系 Relationship of LncRNA AK09398 expression with pathological characteristics of SCLC patients

Clinical characteristics	LncRNA AK09398 expression		*χ*^2^	*P*
	Low	High		
Cases (*n*=118)	52	66			
Age (yr)			0.164	0.685	
< 56	24	28			
≥56	28	38			
Gender			0.008	0.930	
Female	24	31			
Male	28	35			
Disease stage			23.672	< 0.001	
Limited	35	15			
Advanced	17	51			
Lymph node metastasis			16.765	< 0.001	
Yes	20	50			
No	32	16			
Distant metastasis			27.464	< 0.001	
Yes	16	52			
No	36	14			
Smoking			0.014	0.905	
Yes	27	35			
No	25	31			
Chemotherapy reaction			29.801	0.001	
Sensitivity	38	15			
Resistance	14	51			
Survival status			25.451	< 0.001	
Survival	35	14			
Death	17	52			
SCLC: small cell lung cancer.				

### 2.3 LncRNA AK09398的表达与SCLC患者生存分析

采用*Kaplan-Meier*法估计LncRNA AK09398的表达与患者生存时间的关系，结果发现高表达LncRNA AK09398的患者的中位无进展生存时间（progression-free survival, PFS）为9.06个月，较低表达者（19.92个月）缩短，差异有统计学意义（χ^2^=49.260, *P* < 0.001）；低表达LncRNA AK09398患者的中位总生存时间（overall survival, OS）为32.50个月，较高表达者（22.71个月）明显延长，差异有统计学意义（χ^2^=26.700, *P*=0.001）（图 2）。

**2:**
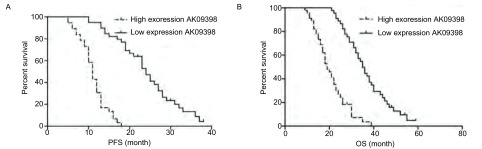
LncRNA AK09398的表达与SCLC患者无进展生存时间及总生存时间的关系。A：LncRNA AK09398的表达与SCLC患者无进展生存时间的关系；B：LncRNA AK09398的表达与SCLC患者总生存时间的关系。 The relationship of LncRNA AK09398 expression with progression-free survival (PFS) and overall survival (OS) in SCLC patients. A: The relationship of LncRNA AK09398 expression with PFS in SCLC patients; B: The relationship of LncRNA AK09398 expression with OS in SCLC patients.

### 2.4 预后影响因素分析

多因素*Cox*回归分析结果显示，LncRNA AK09398的表达、转移及临床分期是SCLC独立的预后因素（表 2）。

**2:** *Cox*多因素分析影响SCLC预后的独立因素 Independent prognosis factors of SCLC by *Cox* multivariate analysis

Characteristics	*β*	S_x_	Wald	RR (95%CI)	*P*
Gender	0.441	0.197	1.008	1.003 (0.635-1.927)	0.550
Age	0.405	0.347	0.494	1.050 (0.427-1.745)	0.970
Disease stage	3.74	2.660	4.040	3.997 (1.850-7.940)	0.001
LncRNA AK09398	4.670	3.830	3.943	5.320 (4.341-11.350)	< 0.001
Distant metastasis	3.240	1.165	2.270	2.530 (1.430-7.160)	0.001
Lymph node metastasis	1.943	1.626	1.979	1.480 (1.173-2.980)	0.003
Chemosensitivity	1.720	0.650	0.470	1.390 (0.946-1.543)	0.070

## 3 讨论

SCLC是一种以生长迅速、早期转移、高度侵袭性为特点的肺癌类型，其发病率约占原发性肺癌的15%-20%^[6]^。由于SCLC肿瘤细胞倍增时间短，进展快，常伴内分泌异常或类癌综合征^[7]^；早期即发生血行转移，故SCLC的治疗应以全身化疗为主，联合放疗和手术为主要治疗手段^[8, 9]^。SCLC的肿瘤细胞早期对化疗和放疗都非常敏感，但几十年来多方案的临床试验并没能找到彻底治愈SCLC的有效方法，因此，寻找SCLC的早期诊断及预后评估标志物已成为目前临床亟待解决的问题。

LncRNA是一类转录本长度超过200 nt的ncRNA，其本身缺乏明显的开放阅读框，不参与蛋白质编码功能，一直被认为是基因转录的“噪音”而未受重视。然而最近的研究^[10]^表明，具有相对较长的核苷酸链，其分子内部具有特定而又复杂的二级空间结构，能提供与蛋白质结合的多个位点，或与DNA、RNA之间通过碱基互补配对原则发生特异性、动态性相互作用，形成由LncRNA参与的基因表达调控网络^[11]^。LncRNA作为近年来新发现的一类调控型非编码RNA成为肿瘤生物学领域中的研究热点，研究发现，LncRNA与肿瘤的形成、浸润、转移过程相关，有望成为新的肿瘤标志物和肿瘤治疗的靶点，在肿瘤诊断和治疗方面显示出良好的临床应用前景。近年来的研究^[12]^发现LncRNA GAPLINC通过靶向作用于SNAI2促进结直肠癌细胞的侵袭性。上调LncRNA NEAT1的表达，促进膀胱癌的进展^[13]^。LncRNA CRNDE的上调与结直肠癌预后差相关，其表达与IRX5 mRNA的表达呈正相关^[14]^；MALAT1是第一个在肺癌中被研究的LncRNA，它可以作为早期肺腺癌中患者预后的一个独立预后标记物^[15]^。HOTAIR在NSCLC样本中高表达，HOTAIR高表达状态与NSCLC淋巴结转移及临床分级相关，此外，HOTAIR高表达的患者预后相对较差^[16]^。CCAT2被发现在NSCLC组织中高表达，CCAT2的高表达水平与肺腺癌相关，提示CCAT2可能是一个肺腺癌特异的LncRNA^[17]^。GAS5在NSCLC组织中低表达，其表达水平与NSCLC的肿瘤大小及临床分级相关。GAS6-AS1在NSCLC组织中低表达，其表达水平与NSCLC的淋巴结转移以及临床分级有关，而且GAS6-AS1是NSCLC患者一个独立预后标记物^[18]^。然而关于LncRNA在SCLC中的功能研究，目前国内外相关报道很少。近年来的研究发现，LncRNA CCAT2促进SCLC的生长和转移，高表达LncRNA CCAT2的患者的预后差^[19]^。LncRNA HOTAIR通过调节*HOXA1*基因的甲基化影响SCLC的化疗敏感性^[20]^。课题组前期通过LncRNA芯片发现LncRNA AK09398在SCLC耐药细胞株中的表达较敏感细胞株中明显增高，提示LncRNA AK09398可能与SCLC的发生发展相关。本研究通过比较118例临床资料完整的SCLC组织及癌旁组织标本中的LncRNA AK09398表达，分析其表达与患者临床病理特征及预后的关系。结果发现LncRNA AK09398在SCLC组织标本中的表达较癌旁组织明显增高。LncRNA AK09398的表达与患者的年龄、性别无关；与疾病分期、淋巴结转移及远处转移、化疗敏感性及生存状态明显相关。高表达LncRNA AK09398患者的总生存时间及无进展生存时间均较低表达者明显缩短；*Cox*多因素回归模型分析提示，LncRNA AK09398的表达、远处转移及临床分期是SCLC独立的预后因素。以上研究结果提示LncRNA AK09398参与调节SCLC的发生发展，可能作为潜在的SCLC预后评估的分子标志物。但LncRNA AK09398在调节SCLC预后中的具体作用机制及参与的信号通路，尚需进行进一步分子实验室及临床研究。
